# Plasma-Based Measurements of Tumor Heterogeneity Correlate with Clinical Outcomes in Metastatic Colorectal Cancer

**DOI:** 10.3390/cancers14092240

**Published:** 2022-04-29

**Authors:** Stephanie J. Yaung, Christine Ju, Sandeep Gattam, Alan Nicholas, Nicolas Sommer, Johanna C. Bendell, Herbert I. Hurwitz, John J. Lee, Fergal Casey, Richard Price, John F. Palma

**Affiliations:** 1 Roche Sequencing Solutions, Inc., Pleasanton, CA 94588, USA; jlee.oscar@gmail.com (J.J.L.); fergal.p.casey@gmail.com (F.C.); palma2112@yahoo.com (J.F.P.); 2 Roche Molecular Systems, Inc., Pleasanton, CA 94588, USA; shen.cju@gmail.com (C.J.); sandeep.gattam@contractors.roche.com (S.G.); 3 Genentech, Inc., South San Francisco, CA 94080, USA; nicholas.alan@gene.com (A.N.); sommer.nicolas@gene.com (N.S.); hurwitz.herbert@gene.com (H.I.H.); price.richard@gene.com (R.P.); 4 Sarah Cannon Research Institute/Tennessee Oncology, Nashville, TN 37203, USA; johanna.bendell@roche.com

**Keywords:** tumor heterogeneity, intratumor heterogeneity, ctDNA, circulating tumor DNA, liquid biopsy, metastatic cancer, colorectal cancer, mCRC, CRC

## Abstract

**Simple Summary:**

Molecular characterization of circulating tumor DNA (ctDNA) can offer a window into tumor genetic heterogeneity, especially in metastatic cancers where different lesions may harbor different mutations. The presence of multiple tumor clones may be reflected by dispersed variant allele frequencies of mutations detected in a single ctDNA sample. We hypothesized that the degree of dispersion of somatic mutations detected with a targeted next-generation sequencing assay may correlate with clinical outcomes in metastatic colorectal cancer. We found that patients with high ctDNA-based tumor heterogeneity after first-line bevacizumab and chemotherapy had shorter progression-free survival and worse objective response. Plasma-based measurements of tumor heterogeneity may have prognostic value in various cancer types and should be further explored for assessing treatment response and other clinical applications.

**Abstract:**

Sequencing circulating tumor DNA (ctDNA) from liquid biopsies may better assess tumor heterogeneity than limited sampling of tumor tissue. Here, we explore ctDNA-based heterogeneity and its correlation with treatment outcome in STEAM, which assessed efficacy and safety of concurrent and sequential FOLFOXIRI-bevacizumab (BEV) vs. FOLFOX-BEV for first-line treatment of metastatic colorectal cancer. We sequenced 146 pre-induction and 89 post-induction patient plasmas with a 198-kilobase capture-based assay, and applied Mutant-Allele Tumor Heterogeneity (MATH), a traditionally tissue-based calculation of allele frequency distribution, on somatic mutations detected in plasma. Higher levels of MATH, particularly in the post-induction sample, were associated with shorter progression-free survival (PFS). Patients with high MATH vs. low MATH in post-induction plasma had shorter PFS (7.2 vs. 11.7 months; hazard ratio, 3.23; 95% confidence interval, 1.85–5.63; log-rank *p* < 0.0001). These results suggest ctDNA-based tumor heterogeneity may have potential prognostic value in metastatic cancers.

## 1. Introduction

Colorectal cancer (CRC) is characterized by high molecular heterogeneity [1,2]. In CRC and other cancer types, analysis of tumor tissue samples, with a single biopsy or multiregional sequencing, can be limited by sampling and therefore not capture genetic heterogeneity within the same sample or between sites [3,4,5,6]. In the past few years, new studies have demonstrated the use of next-generation sequencing (NGS) of circulating tumor DNA (ctDNA) from plasma for molecular characterization of CRC [7,8]. ctDNA sequencing has the potential to reveal more information than tissue sequencing, particularly in the context of therapy resistance beyond the primary tumor [9,10]. Plasma sampling can overcome limitations of tumor biopsy by providing a noninvasive alternative for tumor genotyping and more fully capturing tumor heterogeneity [11].

Various methods have been developed to calculate tumor heterogeneity, though they have been based on whole exome sequencing (WES) results of a single tissue sample per patient and estimates can vary widely by method [12]. Here, we present exploratory analyses of tumor heterogeneity measurements using ctDNA sequencing data from patients with metastatic CRC receiving first-line chemotherapy and bevacizumab in the Sequencing Triplet With Avastin and Maintenance (STEAM) trial. We assessed the potential prognostic value of measuring ctDNA-based tumor heterogeneity with a single plasma sample before and after induction chemotherapy.

## 2. Materials and Methods

### 2.1. Samples and Sequencing

The STEAM study (NCT01765582) was a Phase II trial evaluating bevacizumab (BEV) with concurrent or sequential 5-fluorouracil/leucovorin/oxaliplatin (FOLFOX) or 5-fluorouracil/leucovorin/irinotecan (FOLFIRI) vs. FOLFOX-BEV for the first-line treatment of patients with metastatic CRC. Patients received induction therapy for four months, with an optional addition of two months. Study protocols for STEAM were approved by the Institutional Review Boards at each participating study site and patients provided written informed consent to participate in the biomarker program. Tissue biomarker analysis with KRAS, NRAS, and BRAF as well as final clinical data were previously described [13]. For this analysis, sequencing data were available for 146 pre-induction and 89 post-induction plasma samples. Pre-induction samples were collected one day before or on the day of induction therapy. Post-induction samples were collected 60 to 356 days (median 133 days) after the start of induction therapy. All post-induction samples were collected prior to the start of second line therapy. cfDNA was extracted from 4 mL of plasma and sequenced using the hybrid-capture based AVENIO ctDNA Surveillance Kits (for research use only, not for use in diagnostic procedures) on the Illumina HiSeq 4000. The 198-kilobase AVENIO Surveillance panel covers regions of 197 genes that are recurrently mutated in CRC and lung cancer. The input mass ranged from 0.52 to 208 ng (median 50 ng) and the median deduplicated depth per sample ranged from 504 to 8101 (median 4495).

### 2.2. Variant Calling

Single nucleotide variants (SNVs) and insertion/deletions (indels) were called with the AVENIO ctDNA Analysis Software (for research use only, not for use in diagnostic procedures) [14]. The software includes bioinformatics methods based on CAPP-Seq (cancer personalized profiling by deep sequencing) [15] and iDES (integrated digital error suppression) [16], and reports AF and MMPM values for each detected variant. MMPM is calculated as the AF multiplied by the extracted mass (ng) and adjustment factor of 330 haploid human genome equivalents per ng, then divided by plasma volume (mL). For each sample, the mean AF or MMPM of detected somatic variants was calculated and used as an estimate of tumor content or ctDNA quantity in the plasma.

We differentiated somatic cancer variants from germline variants using a machine learning model [17] that takes into account the variation in allele fraction (AF) from the same mutation found in multiple samples of the same patient. Germline mutations will have stable AF levels over time, whereas somatic ctDNA mutations will have variable AF levels, especially over the course of treatment. In cases where the tumor is not responding to treatment, somatic mutations may also have stable AF levels; therefore, we applied one modification to the method by re-classifying mutations as somatic if they were listed in the Loci of Interest in the AVENIO ctDNA Analysis Software. The Loci of Interest includes variants previously curated for clinical significance in the AVENIO ctDNA Analysis Software [14], and covers cancer hotspots in the following genes in the AVENIO ctDNA Surveillance panel: ALK, APC, BRAF, EGFR, ERBB2, KIT, KRAS, MET, NRAS, PDGFRA, RET, and ROS1.

Given that not all patients had more than one plasma sample available for this analysis, we also applied filters to further remove germline mutations by using public databases of common germline variants. In particular, we classified variants as germline single nucleotide polymorphisms (SNPs) if they were present in >0.001 population frequency in subpopulations in ExAC release 0.3.1 or 1000 Genomes phase3v5b, or annotated as a common SNP in dbSNP build 144.

### 2.3. Calculation of MATH

The formula for Mutant-Allele Tumor Heterogeneity (MATH) is: 100 × MAD/median(X), where MAD is the median absolute deviation, or median(|Xi − median(X)|). MATH for each sample was calculated on the VAFs of somatic variants (Xi) in the sample. For samples with 0 or 1 somatic mutation, a MATH score of 0 was assigned, and these samples were classified as low tumor heterogeneity. Sub-analyses were also performed to separate out these samples as an “undefined” MATH group, as it is possible that additional tumor mutations exist beyond the panel of genes sequenced.

### 2.4. Statistical Analysis

Distributions of patient characteristics were compared between the BEP and the remaining ITT population in STEAM by Wilcoxon rank-sum test, Pearson’s chi-squared test, and Fisher’s exact test as appropriate. Pre and post-induction BEPs were compared with the remaining ITT separately.

PFS was defined as the time from randomization to the date of disease progression or death from any cause in first-line treatment. Subjects without an event were censored based on the last evaluable date of tumor assessment during first-line treatment.

The Kaplan–Meier method and log-rank tests were used to estimate the median PFS and log-rank *p*-value. Unadjusted and adjusted Cox proportional hazards models were used to assess the association between PFS and MATH, either as a continuous or a categorical variable. Unadjusted Cox proportional hazards models were performed for each clinical factor to assess its association with PFS. The clinical variables evaluated were: age, sex, ECOG score at baseline (0 vs. 1), cancer type at diagnosis (colon vs. rectal), location of primary tumor (left vs. right), prior cancer surgery, extent of metastatic disease (liver limited vs. non-liver limited), treatment arm, and liver resection outcome in first line (R0+R1 vs. no resection). Models were assessed separately for pre and post-induction populations. Significant clinical variables (*p* < 0.05) were included in adjusted modeling between MATH and PFS.

ORR was defined as the proportion of patients in first-line treatment with unconfirmed complete response or partial response, following RECIST version 1.1. To assess the association between MATH groups and objective response (complete or partial), logistic regression was used to calculate odds ratio and the associated 95% CI.

Correlations between ctDNA-based metrics such as MATH, mean AF, and number of somatic mutations were assessed by Spearman’s correlation test.

The top 10 most frequently mutated genes, in addition to NRAS, BRAF, and combined RAS status (KRAS or NRAS mutant), were included in the analysis of association between mutated genes and MATH group (high vs. low). Two-sided Fisher exact tests were used for each gene and corrected for multiple comparisons using the Bonferroni method.

## 3. Results

### 3.1. Cohort Characteristics

**Figure A1:**
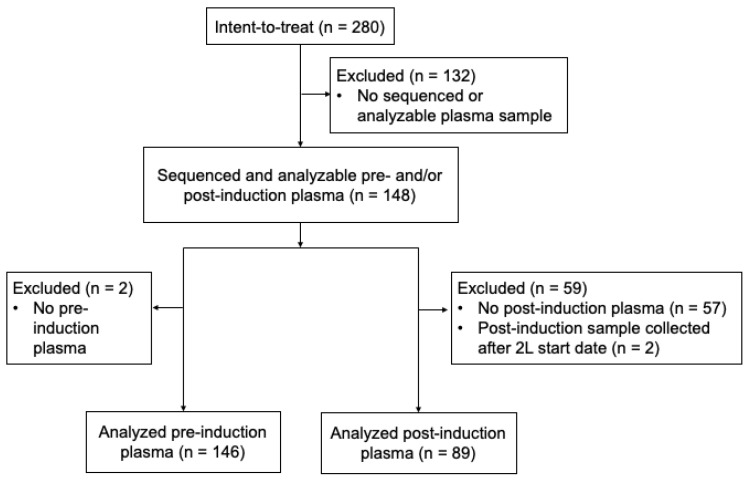
Sample selection for MATH analysis.

**Figure A2:**
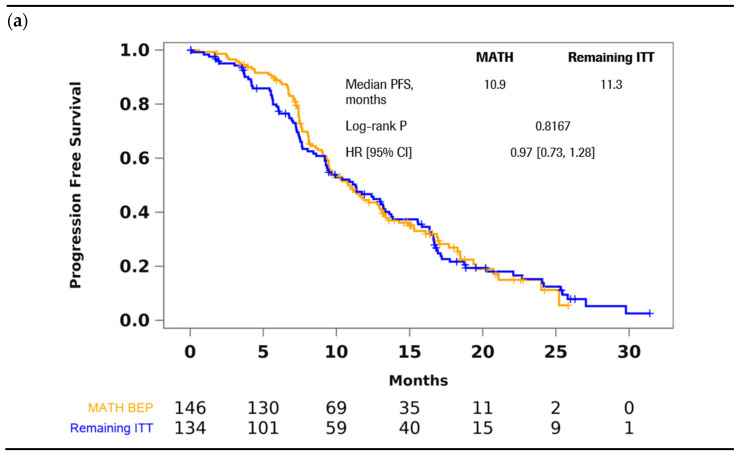

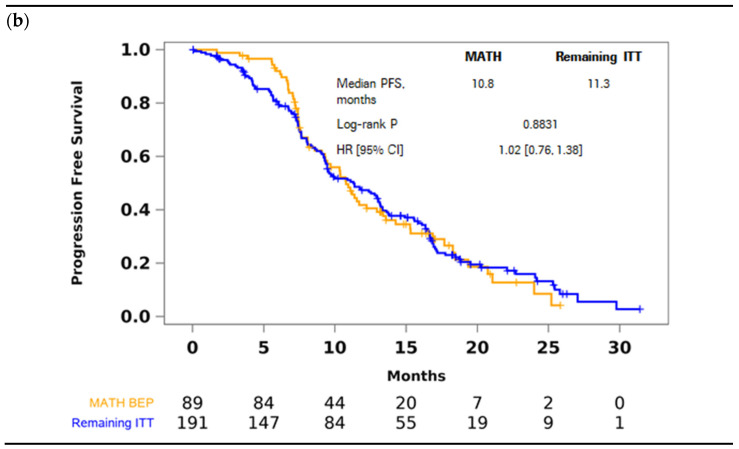
Comparison of PFS with remaining ITT population. (**a**) pre-induction comparison; (**b**), post-induction comparison.

STEAM (NCT01765582) evaluated bevacizumab (BEV) with concurrent or sequential 5-fluorouracil/leucovorin/oxaliplatin (FOLFOX) or 5-fluorouracil/leucovorin/irinotecan (FOLFIRI) vs. FOLFOX-BEV for the first-line treatment of patients with metastatic CRC. Final clinical data have been previously described [13]. Retrospective sequencing on available plasma samples was performed with the AVENIO ctDNA Surveillance Kits (for research use only, not for use in diagnostic procedures). Of the 280 patients in the intent-to-treat (ITT) population in STEAM, 146 had a pre-induction plasma sample and 89 had a post-induction plasma sample available for analysis, which defined the biomarker-evaluable populations (BEPs) (Figure A1). We examined whether patients with evaluable plasma sequencing data had different baseline characteristics compared to the remaining ITT population. Distributions of baseline characteristics were generally not significantly different between the pre-induction or post-induction BEP and remaining ITT population (Table 1 and Table 2). Progression-free survival (PFS) was also not significantly different between the pre/post-induction BEP and remaining ITT populations (Figure A2).

### 3.2. MATH in Pre-Induction Plasma

**Figure 1:**
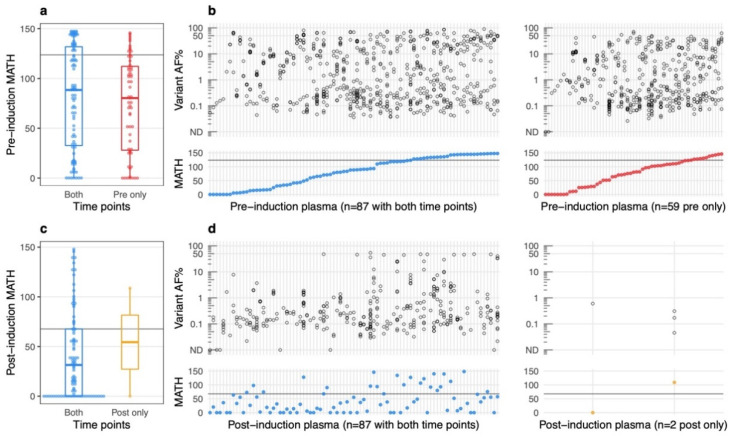
Distribution of plasma-based MATH scores. (**a**) Dot plot and boxplot of MATH measurements for pre-induction plasma samples. Each dot represents a sample. Pre-induction MATH values are not significantly different (*p*-value = 0.2283, Wilcoxon rank-sum test) between patients with only the pre-induction time point (red) and patients with both pre-induction and post-induction time points (blue); (**b**) allele frequency (AF) of detected somatic variants for each sample (top panel). Each open circle represents a somatic variant. The sample’s corresponding MATH score is in the bottom panel. Samples have been ranked in order of increasing MATH score; (**c**,**d**) similar plots shown for post-induction plasma samples. The order of post-induction plasma samples in d matches that of the pre-induction plasma in b for the 87 patients with both time points available for analysis. For all plots, a horizontal line marking the top quartile in pre-induction and post-induction MATH scores is included.

We assessed plasma-based tumor heterogeneity using Mutant-Allele Tumor Heterogeneity (MATH), a measure of variant allele frequency (VAF) variability divided by the median, which was first applied to solid tumors using WES data on tissue samples with matched normal [18]. To determine whether plasma-based MATH could be prognostic of progression-free survival (PFS), we analyzed 146 pre-induction plasma, which were collected either one day prior to or on the day of starting induction therapy. With the AVENIO Surveillance panel, a median of seven somatic mutations (range 0 to 25) were detected per sample. No somatic mutations were detected in two samples, and only one somatic mutation was detected in 11 samples. Figure 1a,b shows the MATH score and respective AF distribution of variants for each sample.

**Figure A3:**
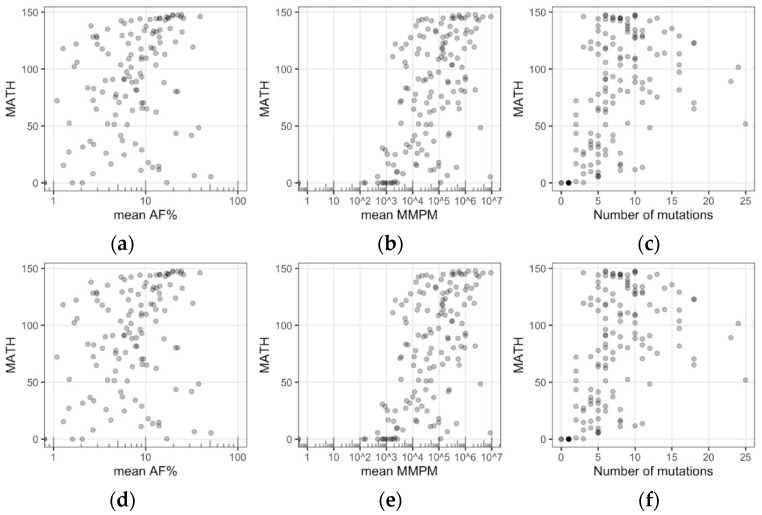
Correlation between MATH and mean AF or mean MMPM or number of somatic mutations. Correlation between pre-induction MATH and mean AF (**a**) Spearman rho = 0.542), mean MMPM; (**b**) Spearman rho = 0.595), or number of somatic mutations; (**c**) Spearman rho = 0.536). Correlation between post-induction MATH and mean AF; (**d**) Spearman rho = 0.493), mean MMPM; (**e**) Spearman rho = 0.489), or number of somatic mutations; (**f**) Spearman rho = 0.809).

As a continuous variable, an increase in pre-induction MATH was associated with shorter PFS (*p* = 0.0128) (Table 3). Given that tumor content in plasma could influence the number of mutations detected and calculations of tumor heterogeneity, the following ctDNA metrics were explored in correlations with MATH and potential adjustment factors: number of somatic mutations detected, mean AF, and mean mutant molecules per milliliter (MMPM). There was moderate correlation between MATH and mean AF (Spearman rho = 0.542, *p* < 0.0001), mean MMPM (Spearman rho = 0.595, *p* < 0.0001), and number of somatic mutations (Spearman rho = 0.536, *p* < 0.0001) (Figure A3a–c). Slight significant association of MATH with PFS was still seen in adjusted models with mean MMPM or number of somatic mutations but not mean AF (Table 3). The association between pre-induction MATH and PFS was also no longer statistically significant (*p* = 0.1341) when adjusting for clinical factors, specifically ECOG, treatment arm, and liver resection during first-line treatment. This was also seen when adjusting for clinical factors with mean MMPM or number of somatic mutations.

**Figure 2:**
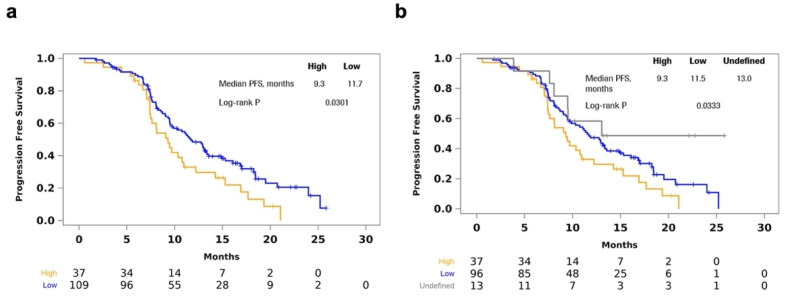
Shorter PFS in high pre-induction MATH. (**a**) Kaplan–Meier curves showing shorter PFS with high tumor heterogeneity, defined as MATH in the top quartile in pre-induction plasma; (**b**) longer PFS with MATH below the top quartile or undefined, i.e., samples with 0 or 1 somatic mutations detected.

Next, we explored MATH as a categorical variable by classifying the top quartile (MATH greater than or equal to 123.9) as high tumor heterogeneity. High tumor heterogeneity was correlated with shorter PFS (median 9.30 vs. 11.70 months; log-rank *p* = 0.0301) (Figure 2a). When samples with 0 or 1 somatic mutation were separated out as an “undefined” MATH group, the difference in PFS was still statistically significant (high vs. low vs. undefined: median 9.30 vs. 11.53 vs. 13.04 months; log-rank *p* = 0.0333) (Figure 2b). However, MATH as a categorical variable (high vs. low) was not significant (hazard ratio (HR), 1.41; 95% confidence interval (CI), 0.91–2.19) when adjusting for ECOG, treatment arm, and liver resection in a multivariable Cox regression model for PFS.

**Figure A4:**
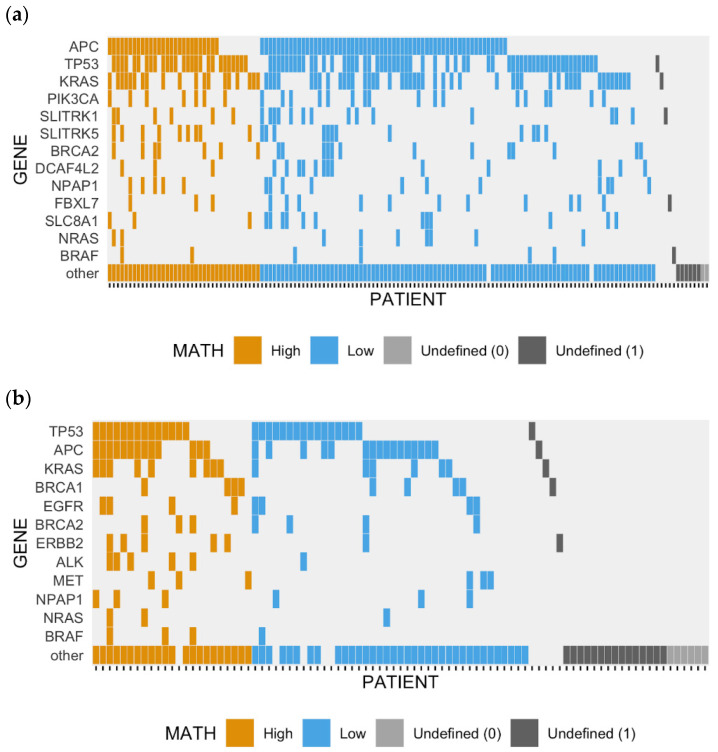
Most frequently mutated genes by MATH group. Mutation landscape for (**a**) pre-induction and (**b**) post-induction plasma. Filled tiles represent mutated genes. Each column represents a patient. Patients are grouped and colored by MATH (High, Low, and Undefined); Undefined are samples with 0 or 1 somatic mutations. The top 10 most frequently mutated genes and NRAS and BRAF are presented. “Other” refers to other genes or no mutations for samples with 0 variants.

Categorical MATH (low vs. high) was also not significantly associated with an objective response in both unadjusted (*p* = 0.8691) and adjusted (*p* = 0.6835) logistic regression models. Of the most frequently mutated genes (Figure A4a), none were significantly enriched in high vs. low MATH groups. KRAS or NRAS were mutated in 54.1% of patients with high MATH and 48.6% of patients with low MATH. BRAF was mutated in 5.4% of patients with high MATH and 4.6% of patients with low MATH. The distributions of RAS and BRAF between MATH low and high groups were not statistically significant (*p* = 0.5681 and *p* = 1.0000, respectively). BRAF mutation in pre-induction plasma was not significantly associated with PFS (HR, 1.44; 95% CI, 0.63–3.31). High vs. low MATH, adjusting for BRAF, was significantly associated with PFS (HR, 1.58; 95% CI, 1.03–2.44). Similar associations were seen for RAS mutation in pre-induction plasma. RAS mutation was not significantly associated with PFS, and high vs. low MATH, adjusting for RAS, was still significantly associated with PFS (HR, 1.61; 95% CI, 1.05–2.47).

### 3.3. MATH in Post-Induction Plasma

We then examined MATH in plasma collected after the start of induction therapy; collection ranged from 60 to 356 days after treatment start date. Eighty-nine patients had a post-induction plasma sample with evaluable sequencing data using the AVENIO Surveillance panel. A median of three somatic mutations (range 0 to 23) per sample were detected. No somatic mutations were detected in six samples, and one somatic mutation was detected in 20 samples. MATH scores and the corresponding distribution of variant AF for each post-induction sample are shown in Figure 1c,d.

Increase in post-induction MATH was associated with shorter PFS (estimated 6% increase in hazard for every five-unit increase in MATH: HR, 1.06; 95% CI, 1.03–1.09) (Table 4). Post-induction MATH was moderately correlated with mean AF (Spearman rho = 0.493, *p* < 0.0001) and mean MMPM (Spearman rho = 0.489, *p* < 0.0001) but strongly correlated with number of somatic mutations (Spearman rho = 0.809, *p* < 0.0001) (Figure A3d–f). However, when including mean AF, mean MMPM, or number of somatic mutations in an adjusted model with MATH, MATH remained significantly associated with PFS (Table 4). The association between post-induction MATH and PFS also remained statistically significant after adjusting for treatment arm and liver resection during first-line (5 unit increase in MATH: HR, 1.05; 95% CI, 1.02–1.08). Association of MATH with PFS was still significant after adjusting for clinical factors with mean AF, mean MMPM, or number of somatic mutations.

**Figure 3:**
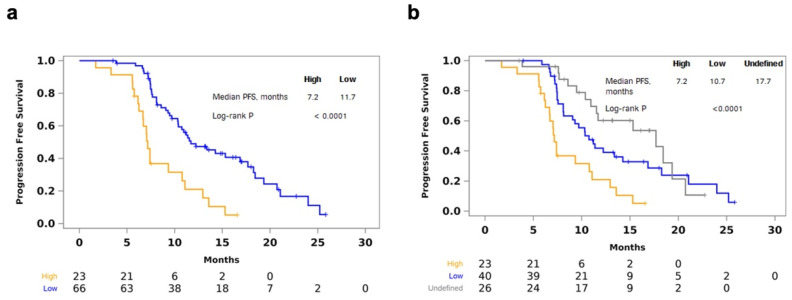
Shorter PFS in high post-induction MATH. (**a**) Kaplan–Meier curves showing shorter PFS with high tumor heterogeneity, defined as MATH in the top quartile in post-induction plasma; (**b**) longer PFS with MATH below the top quartile or undefined, i.e., samples with 0 or 1 somatic mutations detected.

To assess MATH as a categorical variable, we classified post-induction samples in the top quartile (MATH greater than or equal to 67.7) as high tumor heterogeneity. High tumor heterogeneity was associated with shorter PFS (median 7.16 vs. 11.70 months; log-rank *p* < 0.0001) (Figure 3a). When samples with 0 or 1 somatic mutation were designated as a third “undefined” MATH group, the difference in PFS was also statistically significant (median 7.16 vs. 10.74 vs. 17.68 months; log-rank *p* < 0.0001) (Figure 3b). Patients classified in the high MATH category in post-induction plasma had shorter PFS compared to patients classified in the low MATH category (HR, 3.23; 95% CI, 1.85–5.63). MATH (high vs. low) was still significantly associated with PFS after adjusting for mean AF, mean MMPM, or number of somatic mutations (Table 4). The significant association was still observed after adjusting for clinical factors only (HR, 3.34; 95% CI, 1.90–5.86), or in combination with mean AF, mean MMPM, or number of somatic mutations (Table 4).

Objective response rate (ORR) was significantly higher for patients with low MATH compared to high MATH (87.9% vs. 56.5%; *p* = 0.0026) (Table 5). Odds ratio for objective response (complete response or partial response) favored patients with low tumor heterogeneity (OR, 5.58; 95% CI, 1.84–16.88); this was still observed after adjusting for treatment arm and liver resection during the first line (OR, 5.94; 95% CI,1.87–18.90) (Table 5).

Amongst the most frequently mutated genes in post-induction plasma (Figure A4b), KRAS or NRAS were mutated in 43.5% of patients with high MATH and 12.1% of patients with low MATH; this difference was statistically significant after Bonferroni correction (adjusted *p* = 0.033). While patients with RAS mutations in the post-induction plasma had shorter PFS (median difference: 4.3 months; HR, 2.53; 95% CI, 1.42–4.53)), high vs. low MATH status was still significantly associated with PFS in an adjusted model with RAS mutation status (HR, 2.81; 95% CI, 1.58–4.99).

### 3.4. Change in MATH from Pre-Induction to Post-Induction

**Figure 4:**
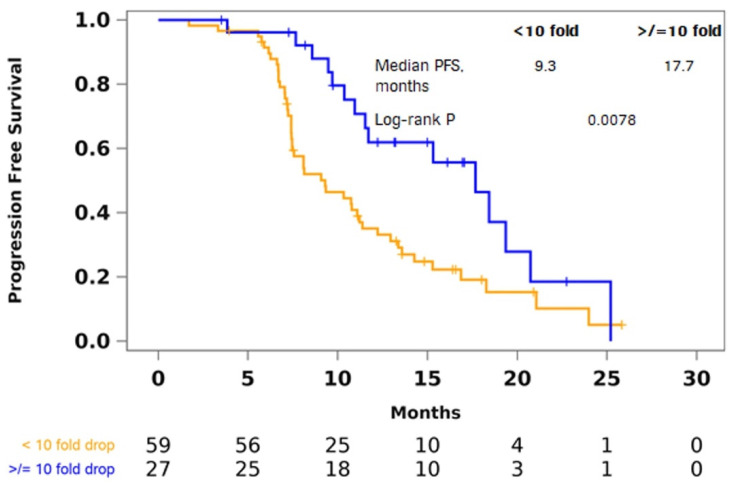
Shorter PFS in patients that did not have a 10-fold decrease. Kaplan–Meier curves showing shorter PFS with a post-induction decrease in MATH from pre-induction not exceeding a 10-fold drop.

**Figure A5:**
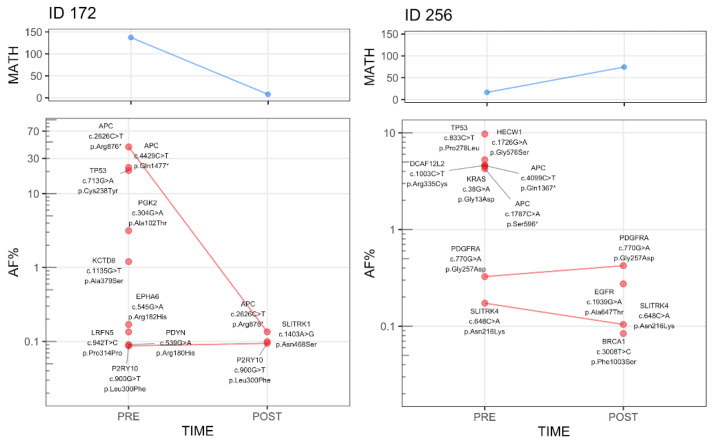
Examples showing variant allele frequencies and MATH. Changes in MATH and AF of individual mutations from pre-induction to post-induction plasma in two cases.

There were 87 patients who had both pre-induction and post-induction plasma samples available for analysis. Out of the 87 patients, four patients had MATH scores equal to 0 at both time points. For these patients, the change in number of somatic mutations was used to determine the MATH classifier. One patient had no change in the number of somatic mutations and was excluded from this analysis. We assessed the change in MATH in the remaining 86 paired plasma samples and found that patients with at least a 10-fold decrease in MATH had longer PFS (log-rank *p* = 0.0078; HR, 2.18; 95% CI, 1.21–3.91) (Figure 4). All three patients with pre and post-induction MATH scores equal to 0 had a decrease of variant count from 1 to 0. These patients were classified in the >/= 10-fold decrease group. The association was no longer significant after adjusting for treatment arm and liver resection during first-line treatment (HR, 1.65; 95% CI, 0.90–3.05) (Table 6). Figure A5 presents examples of changes in VAF in a patient with a greater than 10-fold drop in MATH and another patient with an increase in MATH.

## 4. Discussion

Since sequencing of solid tumor tissue samples may underestimate overall tumor heterogeneity, we hypothesized that heterogeneity could be measured using the distribution of allele frequencies of detected variants from plasma. Using plasma samples collected before and after induction during the STEAM trial, we explored the association of tumor heterogeneity measurements with PFS and ORR. We found that high tumor heterogeneity in post-induction plasma correlated with shorter PFS and worse objective response.

To our knowledge, this is the first application of MATH to ctDNA NGS results, and the first description of plasma-based MATH correlating with clinical outcome. MATH was originally presented as a potential prognostic biomarker in head and neck squamous cell carcinoma [18,19]. Other studies have reported a correlation between MATH and survival in various cancer types, including breast cancer [20], lung adenocarcinoma [21], FGFR3-mutated muscle-invasive bladder cancer [22], melanoma [23], and uterine corpus endometrial carcinoma [24]. Our findings are consistent with studies in CRC that show association of higher MATH scores with poorer outcomes or other clinical and biological factors. For example, higher MATH correlated with a greater number of subclones, which was associated with shorter PFS in CRC Stages I–IV [25]. Higher MATH in male patients in TCGA data was an independent risk factor for shorter OS [26]. MATH correlated with the risk of metastases in stage II CRC [27], and low MATH could predict better response to neoadjuvant chemoradiotherapy in locally advanced rectal cancer [28].

All previous studies with MATH were based on WES of tumor tissue samples. Our work demonstrates feasibility in targeted sequencing of plasma samples, which offers two advantages. First, even though costs of sequencing are declining, WES poses computational and variant interpretation challenges. The 198-kilobase Surveillance panel used in this work was optimized by panel design to maximize the number of mutations that can be detected in CRC and lung cancer. The median number of somatic mutations per patient was 7 and 3 in the pre and post-induction plasma, respectively. Second, liquid biopsy is a non-invasive alternative to tissue biopsy, and can also reflect the genetic heterogeneity of multiple tumor sites that may be undersampled and biased with the sequencing of a single tumor tissue biopsy [29]. Other methods to infer clone phylogeny require collecting multiple tissue samples and face their own analytical challenges [30]. Further studies will be needed to explore plasma-based MATH in other cancer types and treatment regimens.

As for the biological basis underlying the association between plasma-based MATH and response to chemotherapy regimens, more work is required to uncover possible mechanisms. Chemotherapy remains the standard of care for metastatic CRC for patients without targetable mutations even though drug resistance often develops. Chemotherapy is known to apply selective pressure on tumor clones, and ctDNA levels drop during 48-h application of FOLFOX in patients with stable disease or partial response [31]. We observed longer PFS in patients with a greater decrease, such as more than a 10-fold drop, in tumor heterogeneity after the four to six-month induction period. Patients with a smaller decrease, or even stable or increased tumor heterogeneity, may have tumors that have developed resistance to chemotherapy or bevacizumab. Preclinical data show that chronic exposure of CRC cells to bevacizumab leads to increased tumor cell migration, invasion, and metastatic potential [32]. Perhaps tumor cells migrate to other sites and continue to proliferate and evolve new clones, resulting in similar or higher plasma MATH values. Other mechanisms of resistance to bevacizumab treatment are possible. One example is amplification of POLR1D, which may increase expression of VEGFA [33], though we were unable to test this as this region was not sequenced in the panel we used. There may also be tumor evolution through crosstalk with the surrounding microenvironment, where chemotherapeutic agents may upregulate expression of inhibitory immune checkpoints [34,35]. Future studies that incorporate other data types, such as protein or gene expression analysis of immunoregulatory molecules and image-based morphological characterization of tumor heterogeneity [36], could help elucidate these findings.

## 5. Conclusions

Analyses with a 198-kilobase ctDNA NGS assay on the STEAM study enabled the first demonstration of a plasma-based MATH assessment. We found that lower tumor heterogeneity was associated with longer PFS and higher ORR in metastatic CRC treated with first-line chemotherapy and bevacizumab. ctDNA-based tumor heterogeneity may have potential prognostic value in other metastatic cancers and treatment settings.

## Figures and Tables

**Table 1:** Comparison of patient characteristics between pre-induction BEP and ITT.

Characteristic	All Patients (*n* = 280)	Pre-Induction BEP (*n* = 146)	Remaining ITT Population (*n* = 134)	*p*
Age, years (median, range)	57.5 (23–75)	57.0 (23–74)	58.0 (25–75)	0.7534
Sex, *n* (%)				0.9091
Female	118 (42.1)	62 (42.5)	56 (41.8)	
Male	162 (57.9)	84 (57.5)	78 (58.2)	
ECOG performance status, *n* (%)				0.2274
0	165 (58.9)	91 (62.3)	74 (55.2)	
1	115 (41.1)	55 (37.7)	60 (44.8)	
Cancer type at initial diagnosis, *n* (%)				0.9004
Colon cancer	208 (74.3)	108 (74.0)	100 (74.6)	
Rectal cancer	72 (25.7)	38 (26.0)	34 (25.4)	
Prior cancer surgery, *n* (%)	164 (58.6)	81 (55.5)	83 (61.9)	0.2729
Extent of metastatic disease, *n* (%)				0.8501
Liver-limited Disease	83 (29.6)	44 (30.1)	39 (29.1)	
Non-Liver-limited Disease	197 (70.4)	102 (69.9)	95 (70.9)	
Location of Primary Tumor, *n* (%)				0.6969
Left	158 (56.4)	84 (57.5)	74 (55.2)	
Right	122 (43.6)	62 (42.5)	60 (44.8)	
Treatment Arm, *n* (%)				0.8125
FOLFOXIRI/bevacizumab	93 (33.2)	50 (34.2)	43 (32.1)	
Sequential FOLFOXIRI/bevacizumab	92 (32.9)	49 (33.6)	43 (32.1)	
FOLFOX/bevacizumab	95 (33.9)	47 (32.2)	48 (35.8)	
Liver resection rate in 1 L, *n* (%)	18 (6.4)	13 (8.9)	5 (3.7)	0.0779

**Table 2:** Comparison of patient characteristics between post-induction BEP and ITT.

Characteristic	All Patients (*n* = 280)	Post-Induction BEP (*n* = 89)	Remaining ITT Population (*n* = 191)	*p*
Age, years (median, range)	57.5 (23–75)	58.0 (23–74)	57.0 (25–75)	0.5441
Sex, *n* (%)				0.2414
Female	118 (42.1)	33 (37.1)	85 (44.5)	
Male	162 (57.9)	56 (62.9)	106 (55.5)	
ECOG performance status, *n* (%)				0.0257
0	165 (58.9)	61 (68.5)	104 (54.5)	
1	115 (41.1)	28 (31.5)	87 (45.5)	
Cancer type at initial diagnosis, *n* (%)				0.2539
Colon cancer	208 (74.3)	70 (78.7)	138 (72.3)	
Rectal cancer	72 (25.7)	19 (21.3)	53 (27.7)	
Prior cancer surgery, *n* (%)	164 (58.6)	48 (53.9)	116 (60.7)	0.2821
Extent of metastatic disease, *n* (%)				0.6493
Liver-limited Disease	83 (29.6)	28 (31.5)	55 (28.8)	
Non-Liver-limited Disease	197 (70.4)	61 (68.5)	136 (71.2)	
Location of Primary Tumor, *n* (%)				0.4720
Left	158 (56.4)	53 (59.6)	105 (55.0)	
Right	122 (43.6)	36 (40.4)	86 (45.0)	
Treatment Arm, *n* (%)				0.9754
FOLFOXIRI/bevacizumab	93 (33.2)	29 (32.6)	64 (33.5)	
Sequential FOLFOXIRI/bevacizumab	92 (32.9)	29 (32.6)	63 (33.0)	
FOLFOX/bevacizumab	95 (33.9)	31 (34.8)	64 (33.5)	
Liver resection rate in 1 L, *n* (%)	18 (6.4)	8 (9.0)	10 (5.2)	0.2331

**Table 3:** Association of pre-induction MATH with PFS.

Variable	Unadjusted for Clinical Factors		Adjusted for Clinical Factors	
	HR (95% CI)	*p*	HR (95% CI)	*p*
MATH (unit of 5)	1.03 (1.01, 1.05)	0.0128	1.02 (1.00, 1.04)	0.1341
MATH (unit of 5),				
adjusted for mean AF	1.02 (0.99,1.04)	0.1329	1.01 (0.98, 1.03)	0.6691
adjusted for mean MMPM	1.02 (1.00, 1.05)	0.0297	1.02 (0.99, 1.04)	0.0785
adjusted for number of somatic mutations	1.02 (1.00, 1.04)	0.0474	1.02 (0.99, 1.04)	0.1742

Model with MATH with or without adjustment. Clinical factors included were ECOG, treatment arm, and liver resection during first-line treatment.

**Table 4:** Association of post-induction MATH with PFS. Global *p*-values presented for MATH categories with more than two levels (low vs. high. vs. undefined). Clinical factors for adjustment were treatment arm and liver resection during first line.

Model with MATH				
	Unadjusted		Adjusted for Clinical Factors	
MATH Variable	HR (95% CI)	*p*	HR (95% CI)	*p*
unit of 5	1.06 (1.03, 1.09)	<0.0001	1.05 (1.02,1.08)	0.0008
High vs. Low	3.23 (1.85, 5.63)	<0.0001	3.34 (1.90, 5.86)	<0.0001
Low vs. Undefined	1.50 (0.79, 2.85)	0.0001	1.24 (0.65, 2.40)	0.0001
High vs. Undefined	4.14 (2.06, 8.35)		3.83 (1.89, 7.76)	
**Model with MATH and Mean AF**				
	**Adjusted for Mean AF**		**Adjusted for Mean AF and Clinical Factors**	
**MATH Variable**	**HR (95% CI)**	***p***	**HR (95% CI)**	***p***
unit of 5	1.06 (1.03, 1.09)	< 0.0001	1.05 (1.02, 1.09)	0.0009
High vs. Low	3.51 (1.93, 6.39)	< 0.0001	3.69 (2.02, 6.73)	<0.0001
Low vs. Undefined	1.46 (0.76, 2.78)	0.0002	1.18 (0.61, 2.29)	0.0001
High vs. Undefined	4.38 (2.12, 9.08)		4.09 (1.96, 8.50)	
**Model with MATH and Mean MMPM**				
	**Adjusted for Mean MMPM**		**Adjusted for Mean MMPM and Clinical Factors**	
**MATH Variable**	**HR (95% CI)**	***p***	**HR (95% CI)**	***p***
unit of 5	1.06 (1.03, 1.10)	<0.0001	1.06 (1.02, 1.09)	0.0005
High vs. Low	3.54 (1.93, 6.47)	<0.0001	4.25 (2.27, 7.94)	<0.0001
Low vs. Undefined	1.49 (0.78, 2.83)	0.0001	1.19 (0.62, 2.30)	<0.0001
High vs. Undefined	4.50 (2.15, 9.40)		4.72 (2.23, 9.98)	
**Model with MATH and Number of Somatic Mutations**				
	**Adjusted for Number of** **Somatic Mutations**		**Adjusted for Number of** **Somatic Mutations and Clinical Factors**	
**MATH Variable**	HR (95% CI)	***p***	HR (95% CI)	***p***
unit of 5	1.05 (1.02, 1.08)	0.0023	1.04 (1.01, 1.08)	0.0110
High vs. Low	2.78 (1.48, 5.23)	0.0016	3.21 (1.66, 6.20)	0.0005
Low vs. Undefined	1.39 (0.71, 2.74)	0.0037	1.24 (0.63, 2.42)	0.0018
High vs. Undefined	3.58 (1.58, 8.10)		3.76 (1.65, 8.57)	

**Table 5:** Objective response rate by post-induction MATH category. Clinical factors for adjustment included treatment arm and liver resection during first line.

Assessment	Low MATH (*n* = 66)	High MATH (*n* = 23)
ORR (CR or PR)		
*n* (%)	58 (87.9)	13 (56.5)
*p*-value	0.0026	-
Odds Ratio, unadjusted		
Low vs. High (95% CI)	5.58 (1.84, 16.88)	-
*p*-value	0.0023	-
Odds Ratio, adjusted		
Low vs. High (95% CI)	5.94 (1.87, 18.90)	-
*p*-value	0.0026	-

**Table 6:** Association between PFS and decrease in MATH in Cox proportional hazard models. Clinical factors for adjustment included treatment arm and liver resection during the first line.

Model	MATH (<10 Fold vs. >/= 10 Fold Drop)	
	HR (95% CI)	*p*
Unadjusted	2.18 (1.21, 3.91)	0.0093
Adjusted	1.65 (0.90, 3.05)	0.1064

## Data Availability

Data cannot be shared publicly because of legal and ethical restrictions associated with patient confidentiality. Data may be made available to researchers who meet criteria for access to confidential data. For further details on Roche’s Global Policy on the Sharing of Clinical Information and how to request access to related clinical study documents, see here (https://www.roche.com/innovation/process/clinical-trials/data-sharing/ accessed on 28 April 2022).
